# The variation of spike times

**DOI:** 10.1186/1471-2202-13-S1-P132

**Published:** 2012-07-16

**Authors:** Conor Houghton, James B Gillespie

**Affiliations:** 1 School of Mathematics, Trinity College Dublin, Dublin 2, Ireland; 2 Department of Computer Science, University of Bristol, BS8 1UB, UK

## 

Spike trains are often variable with the same stimulus producing different responses from presentation to presentation. These variations can be thought of as being composed of two different types of noise; variations in the spike times and variations in the spike count. The Victor-Purpura distance metric is used to separate these two noise types, allowing the distribution in spike time variations to be calculated. The distribution is calculated for a collection of example data sets. For these data, the distributions are not Gaussian but, in most cases, they can be accurately modeled by a hyper-Laplace distribution.

